# Study on the Status of Health Service Utilization among Caregivers of Left-Behind Children in Poor Rural Areas of Hunan Province: A Baseline Survey

**DOI:** 10.3390/ijerph14080910

**Published:** 2017-08-12

**Authors:** Meimei Ji, Yefu Zhang, Jiaojiao Zou, Tong Yuan, Amber Tang, Jing Deng, Lina Yang, Mingzhi Li, Jihua Chen, Hong Qin, Qian Lin

**Affiliations:** 1 Department of Nutrition Science and Food Hygiene, Xiangya School of Public Health, Central South University, 110 Xiangya Road, Changsha 410078, Hunan, China; jimeimei1024@foxmail.com (M.J.); yefuzhang@foxmail.com (Y.Z.); zjj170605@foxmail.com (J.Z.); yuantong168@foxmali.com (T.Y.); ylnly1997@csu.edu.cn (L.Y.); lmz1976@126.com (M.L.); chenjh@csu.edu.cn (J.C.); 2 Department of Molecular, Cellular and Developmental Biology, Yale University, 219 Prospect St, New Haven, CT 06511, USA; amber.tang@yale.edu; 3 Department of Epidemiology and Statistical Science, Xiangya School of Public Health, Central South University, 110 Xiangya Road, Changsha 410078, Hunan, China; dengjing2@126.com

**Keywords:** health service utilization, left-behind children, caregiver, rural area, China

## Abstract

Background: The caregivers of left-behind children (CLBC) in China’s poor, rural areas are mostly elderly and women. Their health status and access to health services have not been previously characterized. This study aims to explore the status of CLBC in terms of their health service utilization and to provide a scientific basis for guiding effective implementation of health policy in rural Hunan. Methods: Random cluster sampling was used to survey CLBC in two rural counties. Face-to-face interviews and questionnaires were used to collect data, including socioeconomic status and health service utilization. The two-week prevalence rate was used to reflect health service needs, while the two-week visiting rate, annual hospitalization rate and participation in basic public health services were used to evaluate health service utilization. Results: Of the 518 respondents in the study, 95.9% were farmers and 88.4% were over 40 years old. The two-week prevalence rate was 36.1%. Furthermore, 40.1% of ill caregivers’ activities were partly restricted by illness and 3.7% needed to be on bed rest. The two-week visiting rate was 21.0%. The main reasons for not seeing a doctor were “self-medication” (39.1%) or “financial difficulties” (32.6%). The annual hospitalization rate of the CLBC was 22.6% and the non-hospitalization rate of those who needed hospitalization was 41.5%. “Lack of time” (22.3%) and “financial difficulties” (50.5%) were the major factors affecting the utilization of hospitalization services. In terms of participation in basic public health services, only 35.1% CLBC clearly knew that township hospitals have established health records for them. Only 50.6% of caregivers received free health examinations in village clinics or township hospitals and 81.3% of the caregivers did not participate in health education or lectures organized by local health institutions in 2014. Conclusions: The utilization rate of health services was extremely low, which may affect the quality of care for left-behind children. Better public health education through multi-sector cooperation is urgently needed to improve health cognition among CLBC in rural China.

## 1. Introduction

Recent socioeconomic growth in China has led to an increasing population of migrant workers who travel from the countryside to find jobs in nearby cities. This phenomenon has led to the emergence of left-behind children (LBC) who remain in rural areas to be cared for by elderly grandparents, single parents, distant relatives, or neighbors. A national report in 2014 indicated that there were more than 61 million LBC in rural China, accounting for 37.7% of all rural children [1]. The caregivers of left-behind children (CLBC) oversee the daily lives of LBC and are an inevitable social group that has accompanied the appearance of LBC. CLBC in poor, rural areas shoulder both the responsibility of guardianship and a heavy labor burden, placing them at increased risk for poor health outcomes. Given persistent socioeconomic disparities in health, poorer districts have been shown to have lower rates of health care utilization, which is closely related to health status [2]. Furthermore, most caregivers are LBC’s grandparents, who tend to be older and less educated. Their age places them at higher risk for illness and their educational status may affect their health seeking behaviors. The health status of CLBC also directly affects the economic and social status of their families [3].

A previous study in rural China reported low utilization of health services by left-behind elderly and married women. The two-week prevalence rate, two-week visiting rate and annual hospitalization rate of the left-behind elderly was 49.5%, 29.3%, and 30.0%, respectively, in rural Chongqing and 56.9%, 14.9%, and 16.9%, respectively, in rural Hunan. The risk of illness and severity of disease were also higher than that of non-left-behind elderly [4,5]. The corresponding rates for married women were 11.1–14.5%, 5.6–9.4%, and 5.0–6.0%, respectively, in rural areas [6,7,8]. Although these populations of interest may include CLBC, few studies have directly focused on the health service utilization of CLBC. Accordingly, this baseline study examines the health status and health service utilization of CLBC. The objective of this study is to inform future health policy in rural Hunan.

## 2. Methods

This study was conducted as part of a baseline survey of “The impact of conditional cash transfer on the nutritional status and physical development of 3–5 years old left-behind children in poor rural areas of China” [9].

### 2.1. Sampling

Our study took place in the Xiangxi Tujia and Miao Autonomous Prefecture in Fenghuang County, as well as Pingjiang County in Yueyang City. Both counties are identified as poor counties by the government and both are areas densely populated by LBC. Located in the western edge of Hunan Province, Fenghuang County is characterized by a complex landform and is a gathering place of ethnic minorities. Pingjiang County is in the northeastern hilly area of Hunan and inhabited by Han Chinese. These two counties represent the geographical diversity of Hunan Province. 

We used random cluster sampling to recruit 20 eligible villages in each county and 15 LBC households in each village as the target population. The villages and households met the criteria described below:

1. The village inclusion criteria: Villages with a minimum of 15 LBC (3–5 years old) living in poor households (per capita annual income <2300 RMB in 2013) with no kindergarten or care center for LBC.
2. The village exclusion criteria: Villages receiving similar funding or benefits from other sources, such as charities or non-governmental organizations (NGOs).
3. The CLBC inclusion criteria: Caregivers in poor households (per capita annual income <2300 RMB in 2013) with at least one LBC (3–5 years old).
4. The CLBC exclusion criteria: Caregivers receiving benefits from a charity, NGO or other similar program.

### 2.2. Recruitment

Eligible subjects were identified with the assistance of a local village doctor. Caregivers were informed of the study and instructed to go to the village clinic at an appointed time. Informed consent was sought by explaining to potential participants, in a language that they could understand, the goal of the study, the procedure, and the risks and benefits of participation. Individuals were given the opportunity to ask questions following the explanation. If the inclusion criteria were met, caregivers who had signed the informed consent were enrolled in the study.

### 2.3. Ethical Approval

This research was approved by the independent ethics committee of the Institute of Clinical Pharmacology, Central South University and registered in the China Clinical Trial Register (registered number: ctxy-140003). During the investigation, written informed consent by caregivers was obtained and all information was kept strictly confidential.

### 2.4. Data Collection

Face-to-face interviews were conducted by staff at the village clinics or township hospitals. Each staff member was trained by professional investigators from the university. Quality control and guidance personnel were present during the interview process and a trained director conducted further validity checks in order to guarantee the accuracy of the final completed questionnaires.

The baseline investigation was carried out between January and March 2015 to collect data on CLBC household demographics and their health service utilization. The two-week prevalence rate was used to reflect health service needs, while the two-week visiting rate, annual hospitalization rate, and participation in basic public health services were used to evaluate health service utilization. The basic public health service consists of three aspects. First, the township hospitals establish a health record for each resident. Second, residents take an annual, free health examination at their local health institution. Third, residents participate in health education workshops organized by local health institutions.

The two-week prevalence rate is the proportion of the caregivers who developed a disease over a two-week period. The two-week visiting rate is the proportion of ill caregivers who visited a health clinic during a two-week period. The annual hospitalization rate is the proportion of caregivers who were hospitalized in a year. 

The New Cooperative Medical System (NCMS) was implemented in 2003 and was a mutual aid system raised by collectives and individuals in rural China. NCMS aimed to provide low-cost healthcare services for rural residents. The annual per capita payment of NCMS is 90–150 RMB. 

### 2.5. Statistical Analysis

EpiData 3.0 software (The EpiData Association, Odense, Denmark) was used for data entry and the IBM SPSS 18.0 software package (IBM Corp., Armonk, NY, United Sates) was used for data analysis. The statistical methods used in this research include statistical descriptions and chi-squared tests. Descriptive data were reported in the form of a percentage and *p* ≤ 0.05 was considered to be statistically significant.

## 3. Results

### 3.1. General Characteristics of Study Population

A set of questionnaires were administered to 518 CLBC with a valid response rate of 100.0%. Table 1 shows the socio-demographic characteristics of the study population. Most of the caregivers surveyed were female (66.2%), Han ethnicity (62.7%) and farmers (95.9%). Approximately 30% of caregivers had no formal education. Of the respondents, 88.4% were 40 years old and over, 61.6% lived together with their spouses or children, and 83.0% were LBC’s grandparents. Most CLBC household sizes were four to seven members (69.9%), while the most common housing types were brick bungalows (34.0%) and two floor cottages (40.0%). Only 25.9% of caregivers’ households had access to tap water. The average annual per capita income of the sample population was 1512 RMB (The 2013 standard was used in the design of the study. Therefore, the survey results showed that some families’ per capita annual income was higher than the 2013 standard in 2014). In addition, about 96% of caregivers’ households were enrolled in the NCMS.

### 3.2. Accessibility of Health Service

Approximately 80% of caregivers’ families lived within 30 min of the nearest medical institution, 15.8% lived within 31–60 min, and 3.7% lived more than an hour away.

Over a third of households were less than 10 km from the nearest medical institution, a quarter were 21–30 km away, and a fifth were 11–20 km and over 30 km away. Over half of the caregivers walked to medical institutions, 19.6% rode motorcycles, 24.3% took a minibus, and 1.2% rode a bike or a tractor.

### 3.3. Health Service Needs

**Figure 1:**
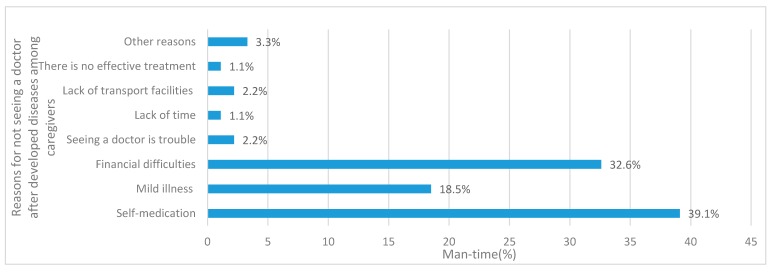
Reasons for not seeing a doctor after developing diseases among caregivers in the past two weeks.

The two-week prevalence rate was used to reflect health service needs. Table 2 shows the two-week prevalence rate was 36.1%, but only 58.3% of ill caregivers reported visiting a health clinic. The main presenting symptoms of illness were fever, headache and cough caused by colds (38.2%), which were accompanied by other symptoms mainly referred to chronic non-communicable diseases. Among ill caregivers, 23.0% experienced two or more symptoms, 41.2% were partly restricted in their daily activities and 3.7% needed to be on bed rest. Table 3 shows village clinics were the primary choice for the CLBC (47.7%). Most caregivers also reported “financial difficulties” (32.6%) or “self-medication” (39.1%) as the main reasons for not seeing a doctor (Figure 1).

### 3.4. Hospitalization Rates

**Figure 2:**
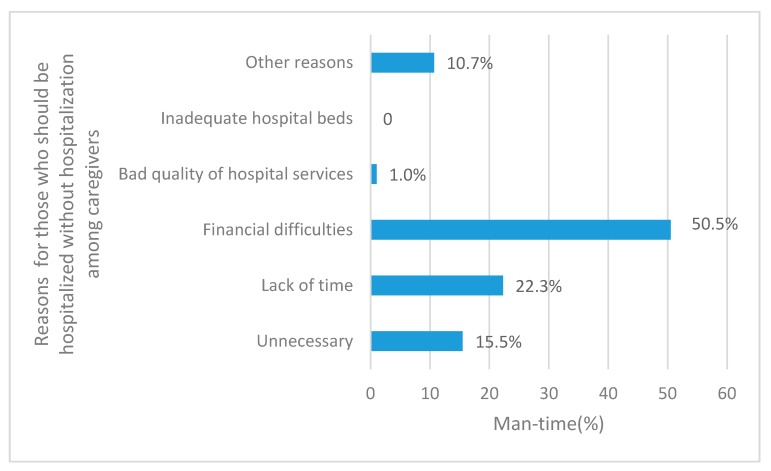
Reasons for those who should be hospitalized without hospitalization among caregivers in 2014.

Over the past year, 117 caregivers were hospitalized (22.6%, 117/518). Table 4 shows that 20.1% of caregivers were hospitalized one to two times because of illness, injury, childbirth, or physical examination and other reasons. Furthermore, 2.5% of the caregivers were hospitalized three times or more, and 78.7% of caregivers stayed in the hospital for six days or more. 41.5% of those who needed hospitalization were not hospitalized. The annual hospitalization rate among CLBC in Pingjiang County (27.2%) was higher than that in Fenghuang County (17.7%) (*p* = 0.035). “Lack of time” (22.3%) and “financial difficulties” (50.5%) were the major factors affecting the utilization of hospitalization services (Figure 2).

### 3.5. Participation in Basic Public Health Services among CLBCs

Table 5 shows that only 35.1% of CLBC clearly knew that township hospitals have established health records for them. Only half of caregivers received free health examination in village clinics or township hospitals in 2014. Furthermore, 81.3% of the caregivers reported that they did not participate in health education or lectures organized by township hospitals or village clinics in 2014. However, the participation rate in health education or lectures in Pingjiang was significantly higher than that in Fenghuang (*p* < 0.001).

## 4. Discussion

Hunan is one of the largest exporting provinces in China with 3.5 million 0 to 17-year-old LBC and 2.9 million 0 to 14-year-old LBC in rural areas in 2014 [1]. It was estimated that LBC consisted of half of all rural children in Hunan. A growing group of CLBC accompanies China’s large LBC population. The present study found caregivers of 3–5-year-old LBC were mainly middle-aged with a low education level. Family income depended on farming and migrant work, which was similar to the findings of another study [10].

The baseline survey showed that the two-week prevalence rate of CLBC was 36.1%, which was higher than 20.2% reported by the Fifth National Health Survey for rural areas [11] and 22.0% reported in a prior study [12]. We also found that 41.2% of ill caregivers’ activities were partly restricted by illness. These findings may be because CLBC are typically older than the general rural population, which could increase the risk and severity of disease.

We found that the two-week visiting rate was 21.0%, which was much lower than 37.2% found by the Fifth National Health Service Survey [11]. CLBC also cited financial difficulties or buying medicine by themselves as the main reasons for not seeing a doctor. This differed from the Fifth National Health Service Survey findings where the major factor was due to mild illness. In a study on health services utilization and influencing factors among rural elderly in poor areas of central and western China [13], surveyed individuals reported that township hospitals were their preferred health facility. However, our study showed the favored medical institutions among caregivers were village clinics, mainly because of proximity. About 80% of caregivers could reach village clinics within 30 min by walking. Meanwhile there was no convenient public transport to the township hospital in the surveyed areas. Although a local private minibus was available to take villagers into town one to two times a week, transportation was not free and there was no fixed schedule. Which greatly affected the caregivers’ choice of medical institutions.

At present, many problems in the infrastructure of rural health facilities in China persist, especially due to a lack of medical instruments and equipment. Village doctors typically have lower educational levels, graduating from junior high school and obtaining relevant qualifications through vocational secondary school [14].Township hospitals and higher health institutions could provide more thorough medical facilities and doctors with higher levels of training, which will aid clinics and hospitals when making definite diagnoses and adopt effective treatment plans. We suggest that it is necessary to improve the training of village health management and health service personnel, in addition to promoting accessibility in poor areas.

We found that 22.6% of CLBCs were hospitalized in the past year, which was about 2.5 times higher than the findings of the Fifth National Health Service Survey. One possible reason is that CLBC were from poor, disadvantaged groups, whose health was typically worse than that of the general rural population. In addition, the annual hospitalization rate among CLBC in Pingjiang County (27.2%) was higher than that in Fenghuang County (17.7%). The geography of Pingjiang is flatter than Fenghuang, which result in improved transportation and distribution of health resources. These factors may account for the higher utilization of inpatient health services in Pingjiang. Our study also found that the non-hospitalization rate of those who needed hospitalization was 41.5% among caregivers, which was much higher than that of 16.7% in rural areas of China [11]. The main reasons for non-hospitalization were “financial difficulties” (43.2%) and “thinking that it was not necessary” (23.7%) in the Fifth National Health Service Survey [11]. In our study, participants reported “financial difficulties” (50.5%) and “lack of time” (22.3%) as the primary barriers to hospitalization. In most cases, economic conditions greatly affected whether or not they were hospitalized. Furthermore, 67.3% of CLBC households had two or more LBC and 21.8% of CLBC households had two or more 3–5-year-old LBC. Given that rural areas lacked centralized care for children and most CLBC worked on the full-time on the farm, CLBC often had to forgo hospital treatment.

In terms of participation in basic public health services, only 35.1% CLBC knew about the established health records at township hospitals. This was far lower than the 76.2% found by the Farmer Health Record Management and Cognition Survey in 2009 [15]. In 2014, only half of caregivers received free health examination in village clinics or township hospitals and less than a fifth of caregivers participated in health education or lectures organized by local health institutions. The participation rate in health education or lectures in Pingjiang County among caregivers was significantly higher than that in Fenghuang County, which could be due to better access to health services and fewer language barriers. Older generations in Fenghuang County could only understand the Miao dialect; however, only a small proportion of medical staff in the region could speak the Miao dialect. Communication barriers might prevent CLBC from attending health lectures. 

Remote and underserved areas in China are more vulnerable to poor health outcomes due to unequal distribution of resources and health services. The Basic Public Health Service Project in China is an important part of promoting the gradual equalization of basic public health services. The project is an important component of China’s medical and health system reform and provides free basic public health services with a focus on children, pregnant women, elderly populations, and those with chronic diseases. The government funds most of the services so that urban and rural residents can directly benefit. However, promoting awareness of these services in rural areas is necessary through broadcasting in the village, placing posters, playing videos in village clinics and township health centers, or holding health lectures. These methods would not only promote health related knowledge among CLBC, but also help publicize resources offered by China’s Public Health Service Project.

Overall, we found that economic factors played an important role in the utilization of health care services among rural caregivers. Although LBC parents find work in the city, it is difficult for them to obtain high-paying jobs due to their low education level [16]. Oftentimes, they are only able to cover their own living costs and the costs of their children’s education. Therefore, migrant workers are not able to fundamentally improve their families’ economic situations.

In view of the current status of health service utilization among CLBC in poor and remote areas of Hunan province, we suggest the following measures. First, strengthen health education efforts to improve health awareness among CLBC. Targeted interventions should be conducted according to specific rural area characteristics. For example, in remote rural areas of Fenghuang County, it is necessary to train healthcare workers in the local language. Medical institutions should also provide health education and promotion materials in the local language. Secondly, bolster basic health infrastructure and raise the level of diagnosis and treatment in village clinics. At the same time, expand access to township hospitals and higher medical institutions. In addition to improving the basic medical insurance system. The NCMS has become an important part of the rural health system and it is the primary means of payment for medical services. While paying close attention to the NCMS participation rate, the Chinese government should also promote awareness of the medical reimbursement system in rural villages. Lastly, reduce the impact of economic factors on health service utilization by improving the implementation of basic public health service projects. 

Health care reforms in other countries may provide a suitable model for improving social services for the elderly. For example, the United States implemented Medicare and Medicaid in 1965, which provide health services for the elderly and low-income [17]. Health care models in developing countries, such as India’s National Rural Health Mission [18] and Thailand’s “30 Baht,” may also serve as examples for China’s future health care reform efforts [19]. In addition to national health care measures, interventions such as Brazil’s Bolsa Familia program, Colombia’s Familias en Acción program, Honduras’ Programa de Asignación Familiar, and Jamaica’s Program of Advancement through Health and Education have also worked to promote health service utilization for low-income populations. These programs work through conditional cash transfer (CCT) payment programs. CCTs are a departure from more traditional approaches of social assistance that represent an innovative and increasingly popular method for the delivery of services. CCTs provide monetary rewards to families contingent upon certain behaviors, such as regular attendance at health centers [20]. The conditionality of CCT provides motivation for the use of health services [21].

Our study is the first to investigate the status of health service utilization among CLBC. These findings are important for guiding effective implementation of health policy. However, our results may be limited by reporting bias as all data were self-reported. In addition, some elderly Miao caregivers could not speak and understand Mandarin and required the use of translators. In this process, some questionnaire items may have been misinterpreted, affecting the accuracy of the results. Nevertheless, our study is novel given its focus on the health status of CLBC. The findings presented here highlight the importance of improving health service accessibility for CLBC in low-income, rural regions of Hunan. Future research is necessary to further explore the factors that affect the utilization of health services and to evaluate whether CCT interventions can improve health service utilization among caregivers.

## 5. Conclusions

The utilization rate of health services was extremely low, which may affect the quality of care for LBC. It is urgent that we invest in rural health services and public health educational programs to improve health service utilization among CLBC.

## Figures and Tables

**Table 1:** Socio-demographic characteristics of study population (n, %).

	Fenghuang County (n = 254)	Pingjiang County (n = 264)	Total (n = 518)
Family size			
≤3	2 (0.8%)	2 (0.8%)	4 (0.8%)
4–7	172 (67.7%)	190 (72.0%)	362 (69.9%)
8–11	73 (28.7%)	67 (25.4%)	140 (27.0%)
≥12	7 (2.8%)	5 (1.9%)	12 (2.3%)
Housing type			
Thatched cottage/shack	37 (14.6%)	4 (1.5%)	41 (7.9%)
Earth wall room	75 (29.5%)	19 (7.2%)	94 (18.1%)
Brick bungalows	73 (28.7%)	103 (39.0%)	176 (34.0%)
Two floor cottages	69 (27.2%)	138 (52.3%)	207 (40.0%)
Access to tap water	104 (40.9%)	30 (11.4%)	134 (25.9%)
Age (year)			
20–39	47 (18.5%)	13 (4.9%)	60 (11.6%)
40–59	91 (35.8%)	152 (57.6%)	243 (46.9%)
≥60	116 (45.7%)	99 (37.5%)	215 (41.5%)
Gender			
Male	81 (31.9%)	94 (35.6%)	175 (33.8%)
Female	173 (68.2%)	170 (64.4%)	343 (66.2%)
Ethnicity			
Han	65 (25.6%)	260 (98.5%)	325 (62.7%)
Miao and other minorities	189 (74.4%)	4 (1.5%)	193 (37.3%)
Education level			
No formal education	99 (39.0%)	56 (21.2%)	155 (29.9%)
Primary school	101 (39.7%)	150 (56.8%)	251 (48.4%)
Junior middle school	44 (17.3%)	48 (18.2%)	92 (17.8%)
High school and others	10 (3.9%)	10 (3.8%)	20 (3.9%)
Occupation			
Farmer	241 (94.9%)	256 (97.0%)	497 (95.9%)
Living conditions			
Living with their spouses	59 (23.2%)	79 (29.9%)	138 (26.6%)
Living with their spouses and children	171 (67.3%)	148 (56.1%)	319 (61.6%)
Widowed and living with their children	21 (8.3%)	27 (10.2%)	48 (9.3%)
Widowed and living alone	3 (1.2%)	10 (3.8%)	13 (2.5%)
Annual per capita income Quartile (RMB)			
<¥695 (Bottom)	84 (33.1%)	45 (17.0%)	129 (24.9%)
¥695–1512 (3rd )	63 (24.8%)	67 (25.4%)	130 (25.1%)
¥1513–2561 (2nd)	56 (22.0%)	74 (28.0%)	130 (25.1%)
≥¥2562 (Top)	51 (20.1%)	78 (29.5%)	129 (24.9%)
Relation to LBC *			
Father or Mother	53 (20.9%)	23 (8.7%)	76 (14.7%)
Grandparents	200 (78.7%)	230 (87.1%)	430 (83.0%)
Others	1 (0.4%)	11 (4.2%)	12 (2.3%)
Bedridden patient at home			
None	215 (84.6%)	206 (78.0%)	421 (81.3%)
One	36 (14.2%)	50 (18.9%)	86 (16.6%)
Two and more	3 (1.2%)	8 (3.0%)	11 (2.1%)
Number of 3–5 years old LBC			
1	193 (76.0%)	208 (78.8%)	401 (77.4%)
2–3	55 (21.7%)	53 (20.1%)	108 (20.8%)
More than 3	3 (1.2%)	2 (0.8%)	5 (1.0%)
Number of participants who had missing data	3 (1.2%)	1 0.4%)	4 (0.8%)
Number of LBC			
1	79 (31.1%)	83 (31.4%)	162 (31.3%)
2–3	139 (54.7%)	160 (60.6%)	299 (57.7%)
More than 3	32 (12.6%)	19 (7.2%)	51 (9.8%)
Number of participants who had missing data	4 (1.6%)	2 (0.8%)	6 (1.2%)
Enrolled in the NCMS **	242 (95.3%)	257 (97.3%)	499 (96.3%)

* LBC: left-behind children; ** NCMS: New Cooperative Medical System.

**Table 2:** Health situation and health service needs among CLBC (n, %).

	Fenghuang County (n = 81)	Pingjiang County (n = 106)	Total (n = 187)	*p*
**Two-week prevalence**	81 (31.9%)	106 (40.2%)	187 (36.1%)	0.055
**Two-week clinic visiting**	44 (54.3%)	65 (61.3%)	109 (58.3%)	0.371
**Presenting symptoms(man-time)**				0.877
Fever/Headache/Cough	36 (39.6%)	48 (37.2%)	84 (38.2%)	
Abdominal pain/ Diarrhea	3 (3.3%)	7 (5.4%)	10 (4.5%)	
Chest pain/Flustered/Palpitations	8 (8.8%)	14 (10.9%)	22 (10.0%)	
Trauma	1 (1.1%)	3 (2.3%)	4 (1.8%)	
Others	43 (47.3%)	57 (44.2%)	100 (45.5%)	
**Types of symptoms**				0.597
One	64 (79.0%)	78 (73.6%)	142 (75.9%)	
Two	14 (17.3%)	20 (18.9%)	34 (18.2%)	
Three or more	2 (2.5%)	7 (6.6%)	9 (4.8%)	
Number of participants who had missing data	1 (1.2%)	1 (0.9%)	2 (1.1%)	
**Is the activity affected?**				0.556
No	48 (59.3%)	55 (51.9%)	103 (55.1%)	
Partly restricted	30 (37.0%)	47 (44.3%)	77 (41.2%)	
Needed to be on bed rest	3 (3.7%)	4 (3.8%)	7 (3.7%)	

Differences were determined by Chi-square test.

**Table 3:** Visits in the hospitals at different levels (n, %).

	Fenghuang County (n = 44)	Pingjiang County (n = 65)	Total (n = 109)	*p*
Village clinics	21 (47.7%)	31 (47.7%)	52 (47.7%)	0.881
Township hospitals	14 (31.8%)	17 (26.2%)	31 (28.4%)	
County hospitals	6 (13.6%)	11 (16.9%)	17 (15.6%)	
City hospitals and above	3 (6.8%)	6 (9.2%)	9 (8.3%)	

Differences were determined by Chi-square test.

**Table 4:** Hospitalization among CLBC (n, %).

	Fenghuang County (n = 254)	Pingjiang County (n = 264)	Total (n = 518)	*p*
**Hospitalized times in 2014**				0.035
None	209 (82.3%)	192 (72.7%)	401 (77.4%)	
One or two times	40 (15.7%)	64 (24.2%)	104 (20.1%)	
Three times or more	5 (2.0%)	8 (3.0%)	13 (2.5%)	
**Total hospital stays in 2014**				0.940
Less than or equal to five days	9 (20.0%)	16 (22.2%)	25 (21.4%)	
Between six and ten days	19 (42.2%)	28 (38.9%)	47 (40.2%)	
More than ten days	17 (37.8%)	28 (38.9%)	45 (38.5%)	
Number of participants who were recommended for hospitalization by a doctor	76 (29.9%)	124 (47.0%)	200 (38.6%)	<0.001
Non-hospitalization for those who needed	31 (40.8%)	52 (41.9%)	83 (41.5%)	0.87

Differences were determined by Chi-square test.

**Table 5:** Received basic public health services among CLBC (n, %).

	Fenghuang County (n = 254)	Pingjiang County (n = 264)	Total (n = 518)	*p*
Have established health records in town hospital				
Yes	83 (32.7%)	99 (37.5%)	182 (35.1%)	0.428
No	101 (39.7%)	103 (39.0%)	204 (39.4%)	
Unclear	70 (27.6%)	62 (23.5%)	132 (25.5%)	
Received free health examination in local health institutions in 2014				
Yes	129 (50.8%)	133 (50.4%)	262 (50.6%)	0.793
No/Forgot	125 (49.2%)	131 (49.6%)	256 (49.4%)	
Received health education organized by local health institutions in 2014				
None	229 (90.1%)	192 (72.7%)	421 (81.3%)	<0.001
One or two times	18 (7.1%)	56 (21.2%)	74 (14.3%)	
Three times or more	7 (2.8%)	16 (6.1%)	23 (4.4%)	

Differences were determined by Chi-square test.

## Abbreviations

The following abbreviations are used in this manuscript:

CLBC	Caregivers of left-behind children
LBC	Left-behind children
NGO	Non-governmental organizations
NCMS	New Cooperative Medical System
CCT	Conditional cash transfer
